# Evaluation of the Immune Effect of a Trivalent Fowl Adenovirus Inactivated Vaccine Against FAdV-4/8a/8b

**DOI:** 10.3390/vetsci12060549

**Published:** 2025-06-05

**Authors:** Yulan Jiao, Qianhui Zhao, Yulong Zhao, Yingjie Li, Sumin Pan, Yinming Li, Yuntao Liu, Wanyu Shi

**Affiliations:** 1 College of Veterinary Medicine, Hebei Agricultural University, Baoding 071001, China; 013307600@163.com (Y.J.); zhaoqianhui2020@163.com (Q.Z.); 2 Ringpu (Baoding) Biological Pharmaceutical Co., Ltd., Baoding 071000, China; 13307600@163.com; 3 Veterinary Biological Technology Innovation Center of Hebei Province, Baoding 071001, China; 4 Veterinary Drug and Feed Work Station of Hebei Province, Shijiazhuang 050011, China; llyj689@163.com; 5 College of Animal Science and Technology, Hebei Normal University of Science and Technology (HNUST), Qinhuangdao 066004, China; pansumin1105@126.com; 6 College of Foreign Language, Hebei Agricultural University, Baoding 071001, China; nmdlym@163.com

**Keywords:** fowl adenovirus, inactivated vaccine, immune protection, neutralizing antibody, HHS, IBH

## Abstract

Fowl adenovirus can cause various diseases such as hepatitis-hydropericardium syndrome (HHS) and inclusion body hepatitis (IBH), which leads to huge economic losses for China’s poultry industry. In recent years, the prevalence of FAdV-4, FAdV-8a, and FAdV-8b has been on the rise. In this study, a trivalent fowl adenovirus inactivated vaccine against FAdV-4/8a/8b was generated and the immune effect was evaluated. The results showed that chickens immunized with the trivalent vaccine could effectively resist the challenge of virulent strains of FAdV-4, FAdV-8a, and FAdV-8b. All chickens in the immunized groups survived after virus injection, gained weight normally, and the lesions in tissues and organs (liver, spleen, kidney, etc.) were significantly decreased compared with the control group. The viral load in the liver of the immunized group was significantly lower than that of the challenge control group five days after the challenge (*p* < 0.01), and the cloacal excretion was significantly lower than that of the non-immune control group (*p* < 0.01). Moreover, until 16 weeks after immunization, the neutralizing antibody level against FAdV-4, FAdV-8a, and FAdV-8b remained above 8.27 log2. As a result, the trivalent vaccine generated in our study was an attractive candidate to prevent and reduce the spread of HHS and IBH in China’s poultry industry.

## Simple Summary

Fowl adenovirus leads to various diseases such as hepatitis-hydropericardium syndrome (HHS) and inclusion body hepatitis (IBH), which induced huge economic losses on the poultry industry in China. In this study, a trivalent fowl adenovirus inactivated vaccine against FAdV-4/8a/8b was generated and the immune effect was evaluated. The results showed that chickens immunized with the trivalent vaccine could effectively resist the challenge of virulent strains of FAdV-4, FAdV-8a, and FAdV-8b, achieving a multi-prevention effect with a single vaccination. This study provides a multivalent inactivated vaccine control method for the fowl adenovirus, which will offer effective immune prevention for FAdV-4, FAdV-8a, and FAdV-8b serotype infections in China.

## 1. Introduction

Fowl adenovirus (FAdV) belongs to the Aviadenovirus genus of the Adenoviridae family [1]. FAdV can be further divided into five species (A, B, C, D, and E) and twelve serotypes (FAdV-1 to 7, FAdV-8a, FAdV-8b, FAdV-9 to 11) [2,3,4,5,6]. FAdV can lead to various diseases in poultry, including inclusion body hepatitis (IBH), hepatitis-hydropericardium syndrome (HHS), and gizzard erosion (GE) [7]. In recent years, FAdV has shown a rapid increasing prevalence worldwide, becoming an important economic disease that affects the poultry industry in Asia, Europe, and other continents [8,9,10,11,12,13,14]. Different serotypes display different tissue tropisms that correlate with clinical manifestations of infection. The predominant serotypes circulating at a given time differ among countries or regions, and change over time, with FAdV-4 and FAdV-8a, 8b being prevalent in Asia [15,16].

Since 2015, as the main symptoms, the cases with IBH and HPS have been prevalent in China on a large scale [17,18,19,20]. The authors have monitored the fowl adenovirus in China in recent years, and the results show that the main circulating FAdV serotypes in China are FAdV-4, FAdV-8a, and FAdV-8b [21]. They primarily infect brooding and growing broiler chicks [22]. The typical characteristics are the sudden death of diseased chickens, accompanied by pericarditis and hepatitis, with a mortality rate as high as 80–100% [20,22]. FAdV-8a and FAdV-8b can lead to chicken inclusion body hepatitis, which often occurs in 3–7-week-old broiler chickens. The mortality rate reaches its peak within 3–4 days after the onset of the disease, ranging from 10% to 30% [10]. Owing to its high infection rate and high mortality rate in broiler chickens, the hepatitis-pericarditis syndrome has caused serious economic losses to the poultry industry [23].

Both domestic and foreign researchers have been working on the development of various vaccines for fowl adenovirus. It has been proven that multivalent vaccines can provide broad-spectrum protection against FAdV [24,25,26]. In accordance with the search results on the China Veterinary Drug Information Network, the fowl adenovirus vaccines currently available in China are mostly FAdV-4 monovalent vaccines or related combination vaccines. These studies, all on FAdV vaccines which are based on FAdV-4, can help prevent HHS, but may not be effective in preventing IBH, as the latter is caused by multiple serotypes [1]. Monovalent vaccines cannot provide cross-protection against heterologous FAdV species [27]. Previous research has shown that FAdV-4 cannot neutralize FAdV-8a and FAdV-8b, and FAdV-8a and FAdV-8b cannot cross-neutralize each other [11]. To prevent IBH effectively, there is an urgent need to develop a safe and effective multivalent FAdV vaccine targeting the currently prevalent serotypes FAdV-4, FAdV-8a, and FAdV-8b in China, in order to control the current HSS and IBH diseases. The purpose of this study is to evaluate the immune protection effect of a trivalent inactivated FAdV vaccine (FAdV-4 + FAdV-8b + FAdV-8a) and provide data support for the subsequent research on multivalent fowl adenovirus vaccines.

## 2. Materials and Methods

### 2.1. Ethical Animal Research

The animal studies were approved by the Animal Care and Use Committee of Hebei Agricultural University (grant no. 2024116). The studies were conducted in accordance with the local legislation and institutional requirements. Written informed consent was obtained from the owners for the participation of their animals in this study.

### 2.2. Virus Strains, Cells and Vaccines

The virus strains used in this study were FAdV-4 (JS strain), FAdV-8a (A10 strain), and FAdV-8b (WS-7 strain). These were provided by Ringpu (Baoding) Biological Pharmaceutical Co., Ltd. (Baoding, China). LMH cells were purchased from ATCC and adapted by Ringpu (Baoding) Biological Pharmaceutical Co., Ltd. The inactivated vaccines of fowl adenovirus FAdV-4 (JS virus strain), fowl adenovirus FAdV-8a (A10 virus strain), fowl adenovirus FAdV-8b (WS-7 virus strain), and the trivalent inactivated vaccine of fowl adenovirus (type 4 JS virus strain + 8a type A10 virus strain + 8b type WS-7 virus strain) (referred to as “trivalent inactivated vaccine” hereinafter) were all provided by Ringpu (Baoding) Biological Pharmaceutical Co., Ltd.(Table 1 for the experimental procedures).

### 2.3. SPF Chicken Embryos, SPF Chickens and LMH Cell

SPF chicken embryos were purchased from Jinan Saisi Laboratory Animal Co., Ltd. (Changsha, China), Shandong Province. SPF chickens were hatched and raised to the required age in the laboratory of Ringpu (Baoding) Biological Pharmaceutical Co., Ltd.

### 2.4. Routine Project Inspection of Inactivated Vaccines

The four inactivated vaccines selected above in part 2.2 were all inspected for vaccine appearance, dosage form, stability, viscosity, sterilizing test, and formaldehyde content in accordance with the current standards of the “Chinese Pharmacopoeia for Veterinary Drugs (Part III)” [28] (hereinafter referred to as “Chinese Veterinary Pharmacopoeia”).

### 2.5. Correlative Analysis of Antigens of Epidemic Virus Strains

Thirty 21-day-old SPF chickens were selected, with 10 in each group. Each group was subcutaneously injected with FAdV-4, FAdV-8a, and FAdV-8b monovalent inactivated vaccines, respectively, at 0.5 mL per chicken. A booster immunization was given 21 days after the initial immunization. Fourteen days after the booster immunization, blood samples were collected and inactivated at 56 °C for 30 min to obtain positive sera for epidemic strains. According to the fixed virus dilution serum method in reference [21], two-way cross-neutralization tests were conducted between the epidemic strains and the corresponding positive sera.

### 2.6. Safety Inspection of Trivalent Inactivated Vaccine

Twenty 14-day-old SPF chickens were selected and divided into two groups, with 10 chickens in each group. The first group was the vaccine immunization group, and each chicken was subcutaneously injected with 1.0 mL of the prepared trivalent inactivated vaccine at the neck. The second group was the negative control group, and each chicken was subcutaneously injected with the same dose of sterilized MARCOL 52 adjuvant white oil at the neck. The clinical signs and feed intake of the experimental chickens were observed every day after inoculation, as was whether there was swelling or other inflammatory reactions at the injection site. After continuous observation for 21 days, the experimental chickens were dissected to observe the absorption of the vaccine at the injection site (Table 2). All the chickens used in the experiment were anesthetized and euthanized in order to alleviate suffering.

### 2.7. Evaluation of the Immune Effect of Trivalent Inactivated Vaccine

Seventy healthy 21-day-old SPF chickens were randomly classified into seven groups, with 10 chickens in each group. According to Table 3, they were divided into vaccine immunization groups, virus infection control groups, and negative control groups. In the vaccine immunization groups, the chickens were subcutaneously injected with trivalent inactivated vaccine at 0.3 mL per chicken in the neck. Fourteen days after immunization, the vaccine immunization groups and the virus infection control groups were injected with the corresponding virus strains, with a virus strain injection dose of 10^7.5^ TCID_50_ for each. The chickens were observed continuously for 14 days post challenge, and the clinical symptoms, morbidity and mortality of each group were recorded. At the same time, the body weight of the chickens in each group was measured on days 0, 3, 5, 7, and 14 post challenge, and cloacal swabs were collected for the detection of fowl adenovirus virus load. Fourteen days post challenge, all the chickens were autopsied to observe gross lesions of tissues and to collect samples of the heart, liver, spleen, lung, and kidney for histopathological observation and fowl adenovirus virus load detection in the tissues and organs (Table 3 for the experimental procedures).

Virus load detection method: Total DNA was extracted from 100 mg tissue samples or 200 μL cloacal swab samples by using a DNA nucleic acid extraction kit. The real-time fluorescent quantitative PCR method developed by GunesA et al. (2012) [13] (primer information is shown in Table 4) was utilized to detect the virus copy amount in the tissues and organs of the experimental chickens and in the cloacal swabs.

### 2.8. Antibody Durability Surveillance in Trivalent Inactivated Vaccines

Twenty 21-day-old SPF chickens were selected and divided into two groups, with 10 chickens in each group. The first group was the immunized group, and each chicken was subcutaneously injected with 0.3 mL of the vaccine at the neck. The second group was the negative control group. Blood samples were collected, and the serum was separated from the experimental chickens at 1W, 2W, 3W, 4W, 8W, 12W, and 16W after immunization. The growth and decline patterns of antibodies were monitored by using the fixed virus dilution serum neutralization test.

### 2.9. Statistical Analysis

Statistical analyses were conducted using lBM SPSS Statistics version 27.0 (lBM Corp., Armonk, NY, USA). All the data were presented as the means ± standard error of mean. The data were analyzed using one-way ANOVA with Duncan’s method for multiple comparisons between groups within the SPSS Statistics software. *p* < 0.05 was considered statistically significant.

## 3. Results

### 3.1. Virus Quality Control

The four selected inactivated FAdV vaccines were subjected to character, sterility and formaldehyde residue tests. The results are shown in Table 4. All inactivated vaccines were milky white emulsions; when the vaccines were dropped into cold water, except for the first drop, the rest did not spread, which indicated that the inactivated vaccines were of water-in-oil (W/O) type; 10 mL of vaccine was taken and added to a centrifuge tube, and centrifuged at 3000 rpm for 15 min. The water phase precipitated at the bottom of the tube did not exceed 0.5 mL, indicating that the inactivated vaccines had good stability; the results of the sterility test and formaldehyde residue determination were in compliance with the current Chinese Pharmacopoeia for Veterinary Drugs.

### 3.2. Cross Neutralization Test

The test results show that the antigens of FAdV-4, FAdV-8a, and FAdV-8b and their respective serum neutralizing antibody titers were 5.0 Log2, 7.0 Log2, and 12.0 Log2, respectively; the antigen of FAdV-4 and serum neutralizing antibody titers of FAdV-8a and FAdV-8b was 0; the antigen of FAdV-8a and the serum neutralizing antibodies FAdV-8b was 2.0 Log2 (Table 5), which indicated that there is no cross-protection among the serotypes of FAdV-4, FAdV-8a, and FAdV-8b.

### 3.3. Safety Controls of Trivalent Inactivated Vaccine

**Figure 1:**
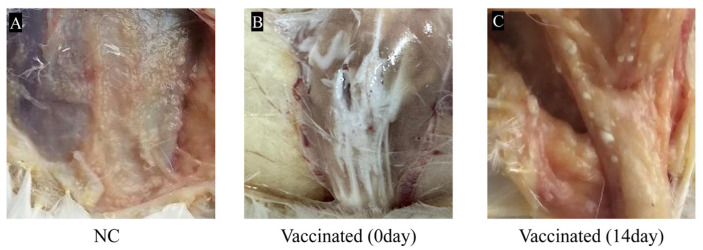
Reactions at the vaccine injection site. (**A**): In the control group, no rotation was observed; (**B**): the vaccine was not absorbed at the injection site on the day of injection; (**C**): After 14 days of vaccination, only a small number of milky white particles the size of small rice grains remained at the vaccine injection site.

The results of the safety inspection showed that both the vaccine-immunized group and the negative control group of chickens experienced temporary depression and reduced feed intake 24 h after injection of the inactivated vaccine and sterilized white oil, respectively. However, after 48 h, the chickens in the experimental group had returned to normal level in terms of spirit, appetite, and vitality. Examination of the injection site showed no redness, swelling, or necrosis. Only a few millet-sized, milky-white granules remained under the skin at the injection site, which was due to incomplete absorption of the white oil and did not affect the use of the vaccine (Figure 1).

### 3.4. Immune Effect of the Trivalent Inactivated Vaccine

#### 3.4.1. The Body Weight Evolution

**Figure 2:**
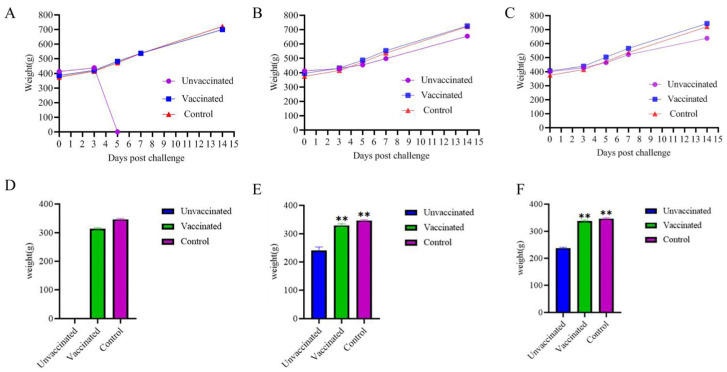
Body weight changes in chickens in the vaccine-immunized group, challenge control group, and control group at 0 d, 3 d, 5 d, 7 d and 14 d after challenge. FAdV-4 (**A**,**D**), FAdV-8a (**B**,**E**), and FAdV-8b (**C**,**F**). The growth weight of chickens in the infection control group was significantly lower than that in the negative control group, while there was no significant difference between the immune group and the negative control group. The difference among all groups was statistically significant (** *p* < 0.01).

The body weights of the vaccine-immunized group, the challenge group, and the negative control group were measured at 0 d, 3 d, 5 d, 7 d, and 14 d after the challenge. The average weight gain of the vaccine-immunized group, the challenge group, and the negative control group at 14 days after the virus challenge was compared. The results showed that the body weight of SPF chickens immunized with the FAdV-4+8a+8b trivalent inactivated vaccine for 14 days was not significantly affected after being inoculated with the corresponding virus strains of FAdV-4, FAdV-8a, and FAdV-8b (Figure 2A–C). The average weight gain of the vaccine-immunized group was analyzed by one-way ANOVA by using SPSS 27.0.1 compared with that of the negative control group, and the difference was not significant (*p* > 0.05). All the chickens in the FAdV-4 infection control group died on the 5th day, so the body weight at 14 days after virus challenge could not be recorded. The body weight of the FAdV-8a and FAdV-8b virus infection control groups decreased significantly compared with that of the negative control group (*p* < 0.01) (Figure 2D,F).

#### 3.4.2. Virus Shedding in Cloacal of Each Group of Chickens

**Figure 3:**
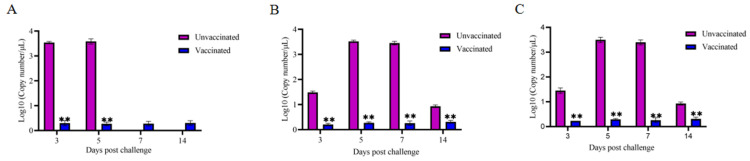
Viral loads in cloacal from chickens in each group after challenging with FAdV-4 (**A**), FAdV-8a (**B**), and FAdV-8b (**C**). A SYBR Green qRT-PCR was used to determine the viral loads in cloacal. The viral loads were calculated as copy numbers/microliter anal swab solution and presented as the mean ± standard error of the mean (** indicate *p* < 0.01).

The virus copy numbers in cloacal excretion samples of the vaccine-immunized group, the virus infection control group and the negative control group were determined by real-time quantitative PCR. The results showed that virus copy numbers in the cloaca of the FAdV-8a and FAdV-8b vaccine-immunized groups were significantly lower than those in the cloaca of the virus infection control group (*p* < 0.01). All the FAdV-4 virus infection control group chickens died on the 5th day, so the cloacal excretion samples on the 7th and 14th days after virus strain injection could not be detected. However, the virus copy numbers in the cloacal of the virus infection control group on the 3rd and 5th days were significantly higher than those of the vaccine-immunized group and the negative control group (*p* < 0.01) (Figure 3).

#### 3.4.3. Virus Load in Organs of Each Group of Chickens

**Figure 4:**
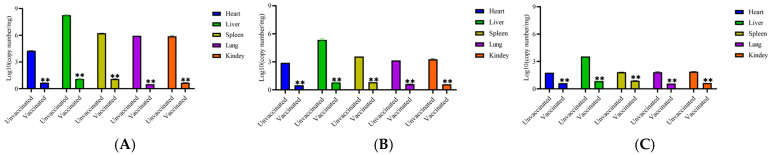
Viral loads in tissues from chickens in each group after challenging with FAdV-4 (**A**), FAdV-8a (**B**), and FAdV-8b (**C**). A SYBR Green qRT-PCR was utilized to determine the viral loads in tissues. The viral loads were calculated as copy numbers/milligram tissue and presented as the mean ± standard error of the mean (** indicate *p* < 0.01).

The virus load in the heart, liver, spleen, lung, and kidney tissue samples of each experimental group of chickens infected with FAdV-4, FAdV-8a, and FAdV-8b were determined. The results showed that (Figure 4) virus loads in the organs of the vaccinated groups were significantly lower than those in the organs of the challenged groups (*p* < 0.01).

#### 3.4.4. Clinical Symptoms and Gross Lesions

**Figure 5:**
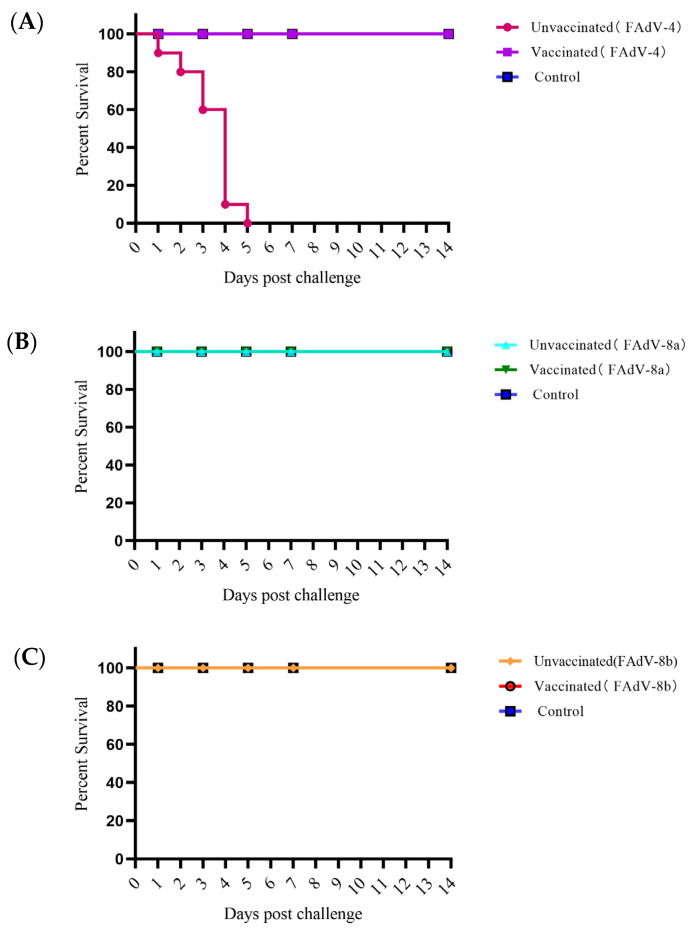
Percent of survival for chickens challenged with virulent FAdV-4, FAdV-8a, and FAdV-8b (**A**–**C**). The challenged chickens were monitored daily for 14 days, and survival rates were calculated.

**Figure 6:**
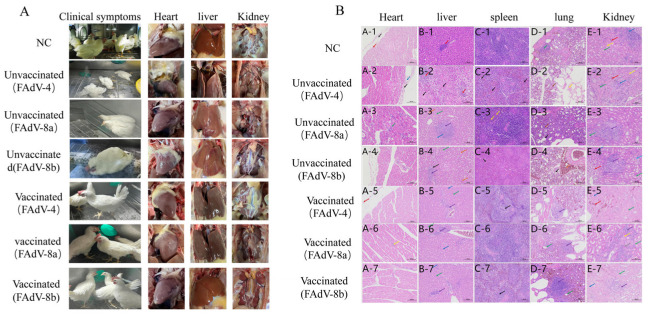
The representative lesion in the heart, liver, and kidney from chickens in each group after challenging with FAdV-4, FAdV-8a, and FAdV-8b. Chickens in the FAdV-4 challenge group showed accumulation of clear straw-colored fluid in the pericardial sac (**A**). The epithelial cells of the renal tubules were denatured and necrotic, with severe degeneration and necrosis of hepatocytes, extensive necrosis of red pulp cells with nuclear fragmentation and dissolution, and degeneration and necrosis of renal tubular epithelium. The FAdV-8a control group exhibited vacuolar degeneration of hepatocytes. The chickens in the FAdV-8b challenge group showed spot-like necrosis, nucleolysis, and eosinophilic red staining of hepatocytes. No histopathological changes were observed in the immunized group or the negative control group (HE staining, original magnification ×200) (**B**).

Clinical symptoms of the chickens in the experimental groups were observed. In the meantime, the hearts, livers, spleens, lungs, and kidneys of the dead and euthanized chickens were collected to observe the pathological changes. The results showed that the chickens in the control groups, which were infected with FAdV-4, FAdV-8a, and FAdV-8b all presented with depression and loss of appetite at 24 h. The chickens in the FAdV-4 virus infection control group began to die at 48 h and all died by 120 h, with a mortality rate of 100% (Figure 5). No deaths were observed in the chickens of the FAdV-8a and FAdV-8b experimental groups and the negative control group. Macroscopic lesions of the chickens in each experimental group revealed that the livers of the virus infection control groups showed various degrees of degeneration and necrosis. Additionally, some of the FAdV-4 infection chickens presented with varying degrees of pericardial effusion, swollen kidneys, and urate deposits. No abnormalities were found in the vaccinated group and the negative control group (Figure 6A).

#### 3.4.5. Histopathological Analysis

The hearts, livers, spleens, lungs, and kidneys of chickens from the negative control group, the challenge control group, and the immunized group were collected for histopathological examination. The results showed that in the FAdV-4 infection control group, extensive hepatocyte necrosis and nuclear fragmentation were observed in the liver group; numerous cells in the red pulp showed necrosis, nuclear fragmentation, and dissolution; a small number of renal tubular epithelial cells showed hydropic vacuolar degeneration. In the FAdV-8a virus infection control group, a small number of hepatocytes showed vacuolar degeneration. In the FAdV-8b virus infection control group, punctate hepatocyte necrosis, nuclear dissolution, and eosinophilic cytoplasmic staining were observed. No obvious abnormalities were found in other organs. In the immunized group and the negative control group, the hepatocytes were round and plump, the hepatic plates were arranged regularly and neatly, the hepatic sinusoids showed no obvious dilation or compression, and no obvious inflammatory changes were observed in the portal areas between adjacent hepatic lobules (Figure 6B).

### 3.5. Antibody Levels in Chicken Immunized with Trivalent Inactivated Vaccine

**Figure 7:**
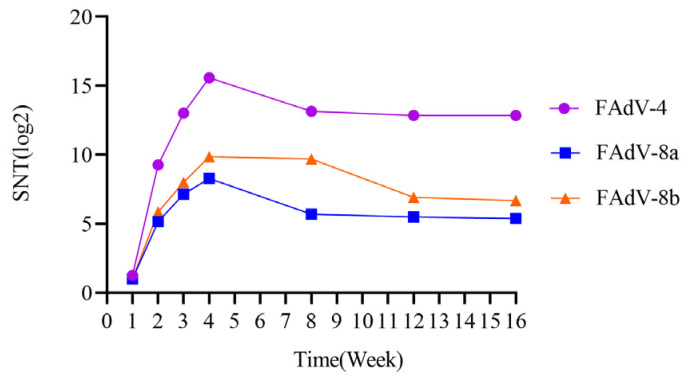
Serum neutralizing antibody levels against FAdV-4, FAdV-8a, and FAdV-8b induced by inactivated vaccines were determined by fixed virus dilution serum neutralization assay.

The serum neutralizing antibody levels against FAdV-4, FAdV-8a, and FAdV-8b caused by the inactivated vaccine were determined. The results showed that the neutralizing antibody titers against FAdV-4, FAdV-8a, and FAdV-8b reached the peak (15.58 log2, 8.27 log2, 9.85 log2) four weeks after immunization with the trivalent inactivated vaccine. The neutralizing antibody titers remained at a relatively high level from 8 to 16 weeks after immunization. The serum neutralizing antibody levels were monitored at the 8th, 12th, and 16th week, and the results showed that the neutralizing antibody titers against FAdV-4 were 13.85 log2, 12.85 log2, and 12.85 log2, respectively; the neutralizing antibody titers against FAdV-8a were 7.93 log2, 7.38 log2, and 7.46 log2, respectively; and the neutralizing antibody titers against FAdV-8b were 9.85 log2, 9.65 log2, and 9.62 log2, respectively (Figure 7).

## 4. Discussion

Fowl adenovirus infection can cause disease in poultry, with the FAdV-4, FAdV-8a, FAdV-8b, and FAdV-11 viruses of the fowl adenovirus being the most pathogenic to domestic fowls [29,30,31]. Moreover, many reports have revealed the occurrence of avian adenovirus infections in China [31,32,33,34]. After infection, these viruses can cause inclusion body hepatitis and pericardial effusion syndrome in poultry. Among them, heart and liver syndrome (HPS) caused by FAdV-4mainly affects 3–6-week-old broilers, with a mortality rate of 30–80%, while inclusion body hepatitis (IBH) caused by FAdV-8a, FAdV-8b, and FAdV-11 can lead to liver necrosis, hemorrhage, and immunosuppression, with a mortality rate of 10–30% [35,36,37]. Outbreaks of fowl adenovirus have brought huge economic losses to the poultry industry. It has been reported that a single outbreak of HPS can result in losses of over USD one million, with an increase in the cost of raising each chicken by USD 0.05–0.1 [38,39].

In early studies, Monreal, G, Kapikian et al. determined the serotypes of fowl adenovirus through serum neutralization tests by using different reference virus strains [40,41,42]. Recently, Steer et al. found that FAdV-8a and FAdV-8b virus strains, both belonging to fowl adenovirus type E, could produce low levels of serum neutralizing antibody titers but showed high levels of neutralizing antibodies against homologous viruses, indicating a certain level of low cross-protection. In this study, the FAdV-4 virus strain and FAdV-8a and FAdV-8b virus strains could not neutralize each other, which is consistent with the results reported by Mazaheri et al. [21]. The antibody titer between FAdV-8a and FAdV-8b was only 1:4, which is consistent with the results of Penelope et al., and was determined to have no cross-protection. Steer PA et al. demonstrated that the neutralizing antibodies produced by a single serotype of a fowl adenovirus vaccine could not cross-protect chicks from infection by heterologous adenovirus species [27]. Recent epidemiological surveys of fowl adenovirus in China have shown that the infection rates of FAdV-4, FAdV-8a, and FAdV-8b are 79.4%, 13.5%, and 3.9%, respectively, and they are the main serotypes in circulation [43,44]. In recent years, China has approved the marketing and application of multiple FAdV-4 fowl adenovirus vaccines, and FAdV-4 has been controlled to a certain extent. However, there are no related vaccines for FAdV-8a and FAdV-8b, and their prevalence is on the rise. The poor cross-protection effect of different serotypes of fowl adenovirus has limited the application scenarios of inactivated vaccines. Therefore, the development of a trivalent inactivated vaccine against the fowl adenovirus (FAdV-4, FAdV-8a, and FAdV-8b) is in line with the current market needs in China.

At present, the types of vaccines against fowl adenovirus include inactivated vaccines (including chicken embryo vaccines and cell vaccines), live vaccines (attenuated vaccines, including chicken embryo vaccines and cell vaccines), subunit vaccines, and recombinant vector vaccines, etc. [45,46,47,48]. However, subunit vaccines only induce low levels of neutralizing antibodies in the body [45], with poor protective effects; the development of recombinant vaccines takes a long time, and the immunogenicity of the vector itself may influence the vaccine effect; although attenuated vaccines can continuously stimulate the body to activate cellular and humoral immunity [47,49], they have the risk of virus shedding and reversion to virulence; vaccines prepared by using chicken embryos have the problems of low antigen content and unstable batch-to-batch potency [50]. In contrast, cell-based vaccines have a high antigen content and can induce the production of high levels of neutralizing antibodies in the body, which results in good immune effects [51]. Inactivated vaccines have a long application history in the prevention and control of fowl adenovirus infections and have achieved significant preventive effects [44,52]. They can induce the production of high levels of neutralizing antibodies in the body and do not have the problems of virus shedding or reversion to virulence.

In this study, the trivalent inactivated vaccines that were developed by using a cell suspension culture process can induce the production of high levels of neutralizing antibodies in the body and effectively resist the attack of strong virulent FAdV-4, FAdV-8a, and FAdV-8b. The main manifestations are that the viral load in various tissues and organs and the virus shedding in the cloaca of the vaccinated group were significantly reduced. The experimental data show that after the vaccinated chicken flocks were injected with FAdV-4, FAdV-8a, and FAdV-8b, the average number of viral genome copies per mg in the heart, liver, spleen, lung, and kidney decreased from 4.27/2.9/1.77 log10, 8.25/5.35/3.54 log10, 6.23/3.58/1.83 log10, 5.95/3.15/1.83 log10, and 5.89/3.28/1.89 log10 in the virus injection control group to 0.67/0.5/0.63 log10, 1.1/0.8/0.85 log10, 1.1/0.83/0.9 log10, 0.51/0.61/0.58 log10, and0.68/0.6/0.64 log10 in the vaccinated group (Figure 4), respectively. The viral load in the cloaca decreased from 3.58/3.52/3.5 log10 in the virus injection control group to 0.27/0.26/0.25 log10 in the vaccinated group (Figure 3). Wang B, Zhao et al. evaluated the immune effect of the inactivated FAdV-4/8b bivalent vaccine, and the viral load in various organs and the virus shedding in the cloaca of the vaccinated group were greatly lower than those in the virus injected group, indicating that the vaccine had good immune effects [53], which is consistent with the results of this study. The above results showed that the immunization with the trivalent vaccine against fowl adenovirus significantly reduced the viral load in the tissues and organs of infected chickens and the virus shedding in the cloaca (greatly reducing the risk of virus shedding into the environment), thereby achieving dual protection for infected and non-infected chickens. This provides a useful reference for the study of the prevention and control mechanism of fowl adenovirus.

The trivalent inactivated vaccine developed in this study can effectively resist the attack of strong virulent FAdV-4, FAdV-8a, and FAdV-8b. Specifically, all the vaccinated chickens survived (Figure 5), and there was no significant weight loss compared with the healthy control group (Figure 2). There were no abnormalities in the liver and other tissues and organs. In the challenge control group, the liver cells of the chickens showed varying degrees of damage (Figure 6): FAdV-4 caused liver cell necrosis, nuclear fragmentation and dissolution; FAdV-8a caused vacuolar degeneration of liver cells; FAdV-8b caused punctate necrosis of liver cells and nuclear dissolution, leading to protein synthesis and bile acid secretion disorders in the chickens and long-term disease consumption, which is an important reason for weight loss. At the same time, in the FAdV-4 challenge control group, many cells in the red pulp of the spleen showed necrosis, nuclear fragmentation and dissolution, and the epithelial cells of the renal tubules showed vacuolar degeneration, leading to systemic infection and death. No obvious pathological changes were observed in the vaccinated group. This is consistent with the results of Wang B et al.’s bivalent inactivated vaccine challenge protection test, where the challenge group showed the above pathological changes in various organs, while the vaccinated group showed no abnormalities [53].

In this study, the neutralizing antibodies against the three serotypes of fowl adenovirus were not less than 8.27 log2 four weeks after vaccination, which indicates that the chicken flocks had a strong ability to resist fowl adenovirus infection. This suggests that neutralizing antibodies play a crucial role in resisting corresponding infections. In addition, the trivalent inactivated vaccine developed in this study induced neutralizing antibodies of 15.58–12.85 log2, 8.27–7.38 log2, and 9.85–9.62 log2 against FAdV-4, FAdV-8a, and FAdV-8b (Figure 7), respectively, 4~16 weeks after vaccination. Combined with the author’s previous research on the prevalence of fowl adenovirus from 2017 to 2022, the onset age of the disease mainly concentrated in 3~11 weeks of age. The immune duration of this vaccine fully covers the entire disease period, providing a basis for the formulation of the vaccine’s immunization program. Gupta A et al. found that the bivalent inactivated vaccine they developed could induce a neutralizing antibody response lasting up to 32 weeks, which also verified the results of this experiment [54]. For breeder and layer chickens, it is recommended to administer 2–3 doses of inactivated vaccines before the onset of lay (at intervals of 3–4 weeks between vaccinations) to ensure sufficiently high antibody levels. This allows maternal antibodies to provide early protection for chicks, thereby reducing the risk of infection during the first week of life. For broiler chickens, a primary vaccination is advised between 7 and 21 days of age. In high-risk areas, a booster vaccination should be administered 3–4 weeks after the primary dose to enhance immunity. Grimes, I.R. Alvarado et al. have demonstrated that maternal antibodies provide protection to offspring against challenges from prevalent strains of avian adenovirus [4,55]. Additionally, Kim et al. found that the FAdV-4 oil-emulsified inactivated vaccine exhibited cross-protective effects in both immunized chickens and their progeny, a conclusion inconsistent with the serum cross-neutralization test results in this study [21]. This discrepancy may be attributed to strain variations. Therefore, further research remains necessary to investigate the field immunization efficacy of vaccines and cross-protection between different serotypes of viral strains.

## 5. Conclusions

A trivalent inactivated vaccine for FAdV-4, FAdV-8a, and FAdV-8b developed in this study can simultaneously resist the infection of FAdV-4, FAdV-8a, and FAdV-8b, achieving a multi-prevention effect with a single vaccination. Four to sixteen weeks after vaccination, the three serum neutralizing antibodies were all higher than 7.38 log2, and the antibody persistence period covered the entire disease cycle. This study provides a multivalent inactivated vaccine control method for fowl adenovirus, which will offer effective immune prevention for FAdV-4, FAdV-8a, and FAdV-8b serotype infections in China.

## Figures and Tables

**Table 1:** Preparation of inactivated vaccines.

Groups	Virulent Strain	Virus Preparation	Vaccine Aqueous Phase Preparation	Preparation of Vaccine Oil Phase	Emulsify and Aliquot the Vaccine
trivalent fowl adenovirus inactivated vaccine against FAdV-4/8a/8b	FAdV-4 (10^7.0^TCID_50_/mL) FAdV-8a (10^7.0^TCID_50_/mL) FAdV-8b (10^7.0^TCID_50_/mL)	The viral suspensions of FAdV-4, FAdV-8a, and FAdV-8b were separately filtered, concentrated threefold, and inactivated with 2% formaldehyde at 37 °C for 24 h, then mixed thoroughly in a 1:1:1 ratio.	Ninety-six parts by volume of inactivation-qualified viral suspension were mixed with 4 parts by volume of sterile Tween-80 in an emulsification tank, followed by agitation until complete dissolution of Tween-80.	Ninety-six parts by volume of mineral oil and 6 parts by volume of Span-80 were homogenized until transparent, sterilized by autoclaving at 121 °C for 30 min, and cooled for storage.	The inactivated vaccine was prepared by emulsifying the aqueous phase and oil phase in a 1:3 ratio, and then stored at 2–8 °C.
fowl adenovirus inactivated vaccine against FAdV-4	FAdV-4 (10^7.0^TCID_50_/mL)	The FAdV-4 viral suspension was filtered, inactivated with 2% formaldehyde at 37 °C for 24 h.			
fowl adenovirus inactivated vaccine against FAdV8a	FAdV-8a (10^7.0^TCID_50_/mL)	The FAdV-8a viral suspension was filtered, inactivated with 2% formaldehyde at 37 °C for 24 h.			
fowl adenovirus inactivated vaccine against FAdV8b	FAdV-8b (10^7.0^TCID_50_/mL)	The FAdV-8b viral suspension was filtered, inactivated with 2% formaldehyde at 37 °C for 24 h.			

**Table 2:** Experimental operating methods for safety inspection.

Group	Test Animal	Quantity	Vaccine Immunity	Criterion
Vaccine immunization group	14 days SPF chickens	10	Trivalent inactivated vaccine prepared by subcutaneous injection into the neck, 1.0 mL/chicken	After 21 days of continuous observation, there was no redness, swelling, necrosis and vaccine residue at the injection site
blank control group		10	Sterile white oil was injected subcutaneously into the neck, 1.0 mL/chicken	

**Table 3:** Evaluation methods for vaccine immunogenicity.

Group	Test Animal	Quantity	Vaccine Immunity	Challenge Experiment	Weigh and Take Cloacal Swabs	Pathological Observation
Vaccine immunization group 1	21 days SPF chickens	10	Trivalent inactivated vaccine prepared by subcutaneous injection into the neck, 0.3 mL/chicken	FAdV-4 strain (RP-C4)	3d, 5d, 7d, and 14d after challenge	After 14 days of challenge, all the chickens were dissected, and the gross lesions of the tissues were observed, and the histopathology and viral load were observed in the heart, liver, spleen, lung, and kidney
Vaccine immunization group 2				FAdV-8a strain (RP-CA)		
Vaccine immunization group 3				FAdV-8b strain (RP-CB)		
Challenge control group 4			-	FAdV-4 strain (RP-C4)		
Challenge control group 5				FAdV-8a strain (RP-CA)		
Challenge control group 6				FAdV-8b strain (RP-CB)		
blank control group			-	-		

**Table 4:** Primers used for qPCR analysis.

Primers	Nucleotide Sequence (5′ → 3′)
FAV-52K-FW	ATGGCKCAGATGGCYAAGG
FAV-52K-RV	AGCGCCTGGGTCAAACCGA

**Table 5:** Cross sera neutralization titer.

Antigen	Serum		
	FAV-4	FAV-8a	FAV-8b
	Neutralizing Antibody Titer (Log2)		
FAV-4	5.0	0.0	0.0
FAV-8a	0.0	7.0	2.0
FAV-8b	0.0	2.0	12.0

## Data Availability

The data presented in this study are available on request from the corresponding author.
